# "All Sorts and Conditions" of Women

**Published:** 1889-03-02

**Authors:** 


### THE DOCTOR'S PULPIT.

### "ALL SORTS AND CONDITIONS" OF WOMEN.

### THE HIGH PRIESTESS OF THE HOME.

TECHNICAL schools and technical training are much studied chapters in the gospel of to-day. The promoters of those institutions proceed upon the assumption that though "an ounce of practice is worth a pound of theory," yet an ounce of practice when combined with sound theory is of greater value than the practice alone. The man who has practiced any particular work will do it better than the unpracticed man, even though he do it unintelligently. But if the work is to reach perfection, it must be done intelligently as well as mechanically. In other words, hands alone are not sufficient for the particular kind of duties Providence has imposed upon man; brains as well as fingers must take part in it all.

There are very few born geniuses in the world; there are, perhaps, none who are born with such a measure of intelligence and handicraft skill as to enable them to do whatever they wish without training. Even the born poet writes boyishly at twenty, and may sing very foolishly at forty. Although he is "born and not made," he would generally be the better for a little making in addition to his birth. The musical geniuses, who astonish the world with their performances on various instruments, have to add to their natural gifts the acquired skill which comes from careful training. The truth is, that for all kinds of duties men and women need intelligent training as well as natural aptitude.

It would be a highly instructive experiment, and might be amusing, to select twenty callings, and to appoint one representative to act as the champion of each in a debating tournament. The domestic matron might represent the wives and mothers. Similarly a spectacled schoolmistress should be advocate for the interesting ladies who devote their lives to literature and learning. A scientific lady-graduate might be spokeswoman for lady doctors, and an iron-lunged female Boanerges for those who prefer the freedom and publicity of the platform. Sick nurses, Post Office clerks, type-writers, and British Museum readers should all have their representatives. Thus a selection might be made of ten women empowered to speak with authority on behalf of the different classes of their sisters. Then let ten men be chosen, each representing a different calling, say a lawyer, a doctor, a clergyman, a butcher, a baker, a candlestick-maker, a farmer, a statesman, and so on. Each should be asked in turn to give his opinion or her opinion as to the absolute and relative importance of his own calling or of her own calling; and then a competent jury of both sexes should decide upon the place to be occupied by every one of the twenty occupations. We do not concern ourselves about the order of all the various professions, but we should be curious to see what position would be assigned to the duty of house and home keeping by such a jury. Would it rank high or low? Would it be among the first half-dozen or nearer the bottom of the scale? Unfortunately, our debating tournament has never been tried, at any rate within historic times; and therefore we can make no appeal for a verdict to those who might have witnessed so interesting an experiment. We can, however, give our own opinion; and that our readers may debate as thoroughly as they please, either with themselves or their friends.

The homely duty of being mistress of a household and female head of a family, ranks among the very highest in order of importance in which man or woman can be engaged. The cultivation of the land or other method of providing food is evidently the most fundamental and indispensable of all man's duties. If this be neglected man can exercise no other function, for he cannot continue in life. Next in order will come, not the statesman's work but that of the teacher of religion and morals. Religion and morality are higher than statesmanship. They are both universal, whereas statesmanship is local, or at most imperial. It is a grave question whether the duties of the statesman are fundamentally and intrinsically more important than those of the mistress of the home and the mother of the family. Motherhood and the rearing of children partake of that indispensable character which belongs to the providing of food. Without food there could be no life, and without motherhood there could be neither statesman nor state. It is also a question whether the character of the state and its people is not really determined in the home. The mother has the ear and heart of her children for the first fifteen or sixteen years of their lives. During the whole period of growth, she, and she almost exclusively, has the management of them, as the gardener has the management of the vine, or of the peach he is nailing to the wall.

The care of children is the chief of all a mother's duties, but it is by no means her only one. She is responsible to a large extent for her husband's health and happiness. Moreover, the eyes of the servants look to the hand of the mistress for guidance, instruction and control. In the present day, too, the sanitary condition of the dwelling, and, therefore, also the physical well-being and even the lives of the various members of the household are almost entirely dependent upon the intelligence and practical ability of the "high priestess of the home." The husband is the provider for the house; the wife is the administratrix of the family resources. The husband is the head of the family to the state and to the world; the wife generally is, and always ought to be, the head of affairs in the home.

But if this be a correct view of the position and responsibilities of a wife and mother, is it not a position, and are they not responsibilities from which any woman, even the most capable, may shrink and turn away? Instead of that, what is the experience of life? That every girl one meets is willing at a moment's notice to enter with a light heart upon such duties and responsibilities. That whilst clergymen, doctors and lawyers spend years of their lives in training for their work; whilst carpenters, builders, tailors and even grocers pass a tenth-part of life in the apprenticeship stage, nobody thinks it the least necessary to provide any special training of any kind for the future mistress of the home. The strange thing is that even men, who see and suffer from the incapacity and want of general training in their wives, take no steps whatever towards establishing a technical school of general domestic economy for young women. Many a man gives far more thought to the choice of a tailor than he does to the choice of a wife. A whim, a momentary impulse, is often sufficient to bring him to a marriage proposal; and if he has the misfortune to be accepted, honour forbids his reconsideration on the morrow, and so his destiny is fixed. Not only are men more or less carefully trained for every conceivable occupation, but even plough horses and costermongers' donkeys have their periods of instruction. But woman, whose destiny is marriage, and whose duties we have ranked with those of the statesman, often receives no more special training than does a sparrow or a fluffy poodle.

Yet we are a clever and a capable people. But we are lacking in scientific rationality. What we *must* do we do, and we generally do it very well. But we do not look around upon our circumstances and affairs with the large, open eye of instructed reason and trained imagination. It is a daily marvel that in spite of their want of training our women attain a fair average of housekeeping efficiency. They have an intense passion for cleanliness, and a natural love for virture; and so the home is generally wholesome and pleasant and the children mostly simple-minded, truthful and good. But if without special training at all they can produce such moderately good results, what might they not be expected to do if they were scientifically taught for three or four years all that appertains to the rearing and training of children, to the care and comfort of husbands, to the management of servants, to domestic sanitation, to household adornment and beauty? The medical profession has been revolutionised by adding to the routine practice which may be picked up by private pupilage a four or five years' course of scientific and systematic training. Similarly wives and mothers might be made incomparably more intelligent and efficient if, in addition to what they learn of domestic management at home, they could be systematically and scientifically taught the general principles which underlie their manifold and profoundly important duties. Our domestic high priestesses, trained by their own and their mothers' experience alone, have proved themselves so worthy that there can be no doubt of their capacity to rise to the very highest regions of excellence. Neither they nor we should be content until they are provided with the fullest opportunities of developing their fine natural powers to their utmost capacity.

